# Sleep quality and levels of stress, anxiety, and depression in patients treated with homeopathy: a prospective study in the Brazilian public healthcare service

**DOI:** 10.1590/1516-3180.2025.3033.06082025

**Published:** 2025-10-27

**Authors:** Fernanda Maria Simões da Costa Fujino, Ana Paula Ribeiro, Denise Castanho Antunes, Renato Jimenez Gomez, Guilherme Eustáquio Furtado, Patrícia Colombo-Souza

**Affiliations:** I Homeopathic doctor at the Hahnemannian George Galvão Institute (IHGG) and the city hall of Guarulhos (SP), Brazil; master’s in Ciências da Saúde at the Universidade Santo Amaro (Unisa), São Paulo (SP), Brazil.; II Physiotherapist; research and internationalization director, and professor of the Programa de Pós-Gradução em Ciências da Saúde at the Universidade Santo Amaro (Unisa), São Paulo (SP), Brazil.; III Occupational therapist and manager of the Multiprofessional Centre for Integrative and Complementary Health Practices (CEMPICS), Guarulhos (SP), Brazil.; IV Nurse and master’s in Ciências da Saúde at the Universidade Santo Amaro (Unisa), São Paulo (SP), Brazil.; V Physical educator and researcher on Sustainability, Physical Activity, Health and Well-being at the Universidade Politécnica de Coimbra, Coimbra, Portugal.; VI Nutritionist and professor of the Pós-Gradução em Ciências da Saúde at the Universidade Santo Amaro (Unisa), São Paulo (SP), Brazil.

**Keywords:** Homeopathy, Sleep disorders, Anxiety, Depression, Stress disorders, Health promotion, Chronic noncommunicable diseases, NCD, Homeopathic treatment, Public health, Sleep quality

## Abstract

**BACKGROUND:**

Chronic noncommunicable diseases (NCDs) have a multifactorial etiology and are associated with psychosocial factors, such as stress, anxiety, and depression. Sleep quality also influences general health and is associated with obesity and NCDs. Homeopathy, as a medical specialty, is effective in managing these conditions because of its comprehensive approach to individuals.

**OBJECTIVE:**

To evaluate the influence of homeopathic treatment on sleep quality and levels of stress, anxiety, and depression.

**DESIGN AND SETTING:**

Observational, longitudinal, and prospective study on individuals over 18 years of age with homeopathic medical follow-up for 6 months in the public healthcare service of Guarulhos, São Paulo.

**METHODS:**

Participants were evaluated initially (T0) and after 3 (T1) and 6 months (T2) using validated questionnaires (Pittsburgh Sleep Quality Index and the Depression Anxiety and Stress Scale), following all ethical precepts. The scores were compared over time and correlated with each other (P < 0.05).

**RESULTS:**

The mean patient age was 49 years. Initially, 81% of the participants had sleep disorders and severe or extremely severe levels of stress (33.78%), anxiety (28.38%), and depression (27.03%). A total of 26 patients were present at the three evaluation points, which were included as the participants of the study. Homeopathic treatment significantly improved sleep quality and reduced stress, anxiety, and depression. Sleep quality and anxiety were strongly (r = 0.53, P = 0.005) and weakly (r = 0.25, P = 0.021) correlated with stress, respectively.

**CONCLUSION:**

In the short term, homeopathic treatment had a positive impact on the sample, suggesting that this therapy can be used to prevent NCDs.

## INTRODUCTION

 Currently, the main causes of mortality and high healthcare costs worldwide are chronic noncommunicable diseases (NCDs), which are characterized by multiple etiologies, many risk factors, long latency periods, prolonged courses, non-infectious origins, deficiencies, and functional disabilities.^1,2^

 According to the World Health Organization (WHO), NCDs, including cardiovascular diseases, neoplasms, chronic respiratory diseases, and diabetes mellitus, are the main causes of mortality and high healthcare costs globally.^3^ Factors such as unhealthy lifestyles, diet, insufficient physical activity, tobacco and alcohol consumption, and psychosocial factors, including high levels of anxiety, stress, and depression, are crucial determinants in the pathogenesis of these diseases.^1,4^

 Sleep quality is fundamental to general health and is directly associated with the incidence of obesity and several chronic diseases.^5^ Inadequate sleep can aggravate mental health conditions like stress and depression, creating a vicious cycle that worsens NCDs.^6^ Improving sleep quality and reducing symptoms of stress, anxiety, and depression are essential for the prevention and control of NCDs.^5,6^

 Homeopathy, a medical specialty with a comprehensive approach to the individual, considers the complexity and individual needs of each patient and is particularly effective in managing multifactorial conditions such as NCDs. It addresses all physical, emotional, mental, and behavioral signs and symptoms with the aim of promoting the integral state of balance of the organism.^7,8^ Thus, it contributes to the prevention and control of NCDs and can consequently help reduce mortality and healthcare costs associated with these diseases. Furthermore, the homeopathic approach is recognized and legitimized by the National Policy on Integrative and Complementary Practices (PNPIC) as a viable and accessible option for the general population and is offered in Brazilian public healthcare services.^9^

 Considering the above, the objective of this study was to evaluate the influence of homeopathic treatment on the quality of sleep and levels of stress, anxiety, and depression in patients treated by the public healthcare service in the city of Guarulhos, São Paulo. 

## METHODS

 This was an observational, longitudinal, and prospective study carried out with patients over 18 years of age, regardless of gender, who began homeopathic medical follow-up from June 21, 2022, to January 31, 2023, in two healthcare services in the city of Guarulhos. Convenience sampling was performed based on the inclusion criteria. 

 In addition to routine homeopathic appointments, which consisted of homeopathic anamnesis, diagnosis of the situation presented by the individual, prescription of homeopathic medicine, and guidance regarding lifestyle habits, specific and validated questionnaires were also applied for the studied variables in the first appointment and in the follow-up appointments at 3 and 6 months after the initial assessment. 

 Only patients treated by the same homeopathic doctor in the two healthcare services in the city that offered homeopathic care were considered for inclusion in the study. This doctor was the main study researcher who conducted the questionnaires at all times during the evaluation. The individuals had homeopathic medical follow-up appointments for 6 months, with an assessment of their evolution at three time points: T0, time zero (first appointment); T1, time one (3 months of follow-up); and T2, time two (6 months of follow-up). 

 For sleep analysis, the Pittsburgh Sleep Quality Index (PSQI) was applied.^10-12^ This questionnaire consists of 19 items grouped into seven components, each scored on a scale from 0–3. The scores of the seven components were added together to obtain an overall PSQI score ranging from 0 to 21. Scores of 0–4 indicate good sleep quality, 5–10 indicate poor sleep quality, and > 10 indicate sleep disorders. 

 The patient’s emotional state was assessed using the Depression Anxiety and Stress Scale, short version of 21 items (Depression Anxiety and Stress Scale: DASS-21).^13^ The DASS-21 is composed of a set of three Likert-type subscales (depression, anxiety, and stress). Each subscale consists of seven items designed to assess the emotional states regarding depression, anxiety, and stress. Individuals were asked to answer questions based on the week prior to the assessment. Four response possibilities of severity or frequency were provided, organized on a scale of 0–3 points, with the results obtained by adding up the responses to the items that make up each of the three subscales.^14,15^ According to the final score per subscale, an individual’s level of stress, anxiety, and depression was assessed at that particular moment, as shown in Table 1. 

**Table 1:** Score interpretation determined by each subscale after applying the Depression Anxiety and Stress Scale, short version of 21 items (DASS-21)

**Score**	**Stress level**
0–10	Normal (N)
11–18	Low (L)
19–26	Moderate (M)
27–34	Severe (S)
35–42	Extremely severe (ES)
**Score**	**Anxiety level**
0–6	Normal (N)
7–9	Low (L)
10–14	Moderate (M)
15–19	Severe (S)
20–42	Extremely severe (ES)
**Score**	**Depression level**
0–9	Normal (N)
10–12	Low (L)
13–20	Moderate (M)
21–27	Severe (S)
28–42	Extremely severe (ES)

 It is worth mentioning that at T1 and T2, patients were asked whether they used any other medication and whether they sought out any form of treatment or other therapies due to issues related to anxiety, stress, discouragement, sadness, or problems with sleep during the study period, with the aim of ensuring that there had not been any stimulus of this nature since the beginning of the evaluation. If positive, the individual was excluded from the final analysis. 

 Individuals took part in the research after signing a Free and Informed Consent Form containing all study information in a clear and detailed manner. Data collection began only after approval from the Research Ethics Committee of Universidade Santo Amaro (Unisa) under Opinion 5.469.720. 

 Analysis of variance (ANOVA) was applied to compare the scores at the initial time (T0) and at 3 and 6 months after the start of the homeopathic treatment (T1 and T2, respectively), with reference to sleep quality and levels of stress, anxiety, and depression in the studied individuals. Pearson’s correlation was performed to evaluate the degree and direction of the linear relationship between sleep quality and stress, depression, and anxiety scores, considering 0.05 or 5% for the rejection level of the null hypothesis. 

## RESULTS

 Initially, 74 patients were evaluated, the majority of whom were women (89.19%) aged between 35 and 63 years (average 49 ± 13.97 years). At T0, 81% of patients had a change in their normal sleep pattern (poor sleep or sleep disorder), and a significant proportion had severe or extremely severe levels of stress, anxiety, and depression (33,78%, 28,38% and 27,03%, respectively). 

**Figure 1:**
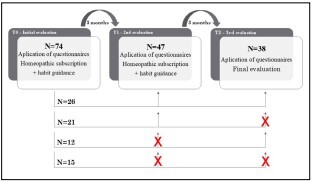
Sample loss during the observation of patients’ evolution after the start of homeopathic treatment in the public healthcare service of Guarulhos (SP).

 Of the 74 patients evaluated initially, 26 returned in 3 (T1) and 6 (T2) months (Figure 1), making these individuals the focus of our study. 

**Figure 2:**
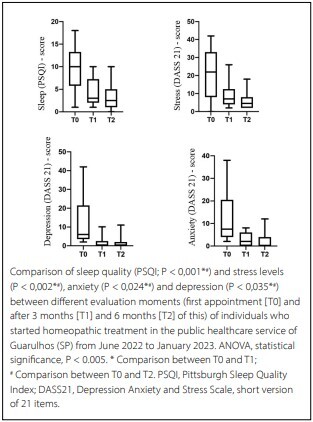
Positive impact of treatment on sleep quality, stress level, anxiety and depression.

 In Figure 2, the scores obtained by applying the questionnaires at the first appointment, at 3 (T1) and 6 months (T2) after the start of the homeopathic treatment were compared regarding sleep quality and the levels of stress, anxiety, and depression in the 26 patients who were present at the time of the three assessments. 

Table 2 shows the correlation of sleep quality (poor or sleep disorder) with stress, anxiety, and depression levels after 6 months of homeopathic treatment (T2). There was a strong positive Pearson’s Correlation (r = 0,53/P = 0,005*) between sleep changes and anxiety, and a weak correlation (r = 0.25/P = 0.021*) between changes in sleep disorders and stress level. 

**Table 2:** Correlation between sleep quality and levels of stress, depression, and anxiety of individuals attended by the public healthcare service in Guarulhos (SP) after 6 months of homeopathic treatment (T2)

**Variable (score at T2)**	**Mean ± SD**	**Sleep quality (score at T2)**	**r**	**P**
Level of depression	1,3 ± 0,9	3,6 ± 2,8	0,03	0,878
Level of anxiety	2,4 ± 2,1	3,6 ± 2,8	0,53	0,005*
Level of stress	6,2 ± 4,9	3,6 ± 2,8	0,25	0,021*

Pearson’s Correlation Test.

* Statistical significance P < 0.005.

## DISCUSSION

 The importance of sleep duration and quality of health is widely evidenced in studies that have found an association between poor sleep quality and the appearance of cardiovascular diseases (CVDs) and metabolic, respiratory, mental, and musculoskeletal diseases.^16^ While some studies have identified the impacts of morbidities on sleep quality,^17,18^ others suggest that sleep deficiency and disorders can develop pathological processes and lead to diseases. Evidence in this regard is consistent with that of CVDs.^16,19,20^

 In this study, the observation of poor sleep or sleep disorders among the patients during their first appointment was noteworthy, and this finding was a risk factor for NCDs in the studied population. During the initial assessment, a significant proportion of the individuals experienced severe or extremely severe levels of stress, anxiety, and depression. Because these conditions are involved in the pathogenesis and incidence of NCDs,^21^ especially those related to CVDs, it is presupposed that this situation is another risk factor for NCDs in this sample. 

 In recent decades, epidemiological studies have demonstrated strong evidence of an association between depression and CVDs.^22^ The cardiotoxic effects of depressive symptoms have been consistently observed, which is why it is important to invest in strategies to prevent this emotional state, in the same way that stressful situations must be tackled, as there is evidence that daily exposure to chronic stress or severe psychological trauma can also increase the risk of developing and dying from CVDs.^22,23^

 Stress is associated with a greater risk of hypertension, acute myocardial infarction, arrhythmogenesis, and heart failure.^21,22^ This is because changes occur in the body in response to chronic stress experienced daily owing to biological mechanisms that may be responsible for elevated hypothalamic-pituitary-adrenal axis activity, reactivity of the autonomic nervous system, inflammation, oxidative stress, and endothelial dysfunction, which may be associated with the development of cerebrovascular diseases and CVDs.^22,23^

 Likewise, anxiety or anxiety disorders can influence the onset or progression of CVDs, due, for example, to the fact that anxiety is associated with unhealthy behaviors, such as tobacco consumption, excessive alcohol intake, lower physical activity, and poor diet, which increase the risk of CVDs.^24^ In the same way as chronic stress, the organism reacts to the stimulus caused by anxiety through the excessive activation of the hypothalamic-pituitary-adrenal axis and the sympathetic nervous system, increasing the release of plasma catecholamines, resulting in endothelial damage, ultimately leading to atherosclerosis, coronary artery disease and possible acute coronary events, making it important to identify these risk factors and develop mechanisms for their prevention.^21-24^

 The homeopathic approach to mental conditions is well documented in literature, which meets the need to use tools that help in the psycho-emotional balance of individuals, avoiding the development of situations that further compromise their health.^25^ To exemplify the role of homeopathy in this context, we can mention the study carried out in 2008 that reports a series of clinical cases of depression treated exclusively with homeopathy at the Outpatient Clinic of Homeopathy and Depression of the public healthcare service in Jundiaí, São Paulo, showing a therapeutic response, with a reduction of more than 50% in depression scores in 93% of patients after seven weeks of treatment, on average, suggesting that homeopathy can be a therapeutic alternative in the treatment of depression.^24^

 In 2010, Giorgi and collaborators^26^ showed that homeopathic treatment resulted in a higher rate of anxiety reduction after 90 days of treatment compared to conventional anxiolytics, in addition to no side effects, which occurred with allopathic medicines traditionally used to control anxiety. Two groups were analyzed: one group was treated with diazepam (benzodiazepine) and the other with homeopathic medicine, with a greater reduction in anxiety in the group treated with homeopathy. Zepeda-Quiroz et al.^25^ also showed an excellent response in the management of anxiety and depressive disorders with homeopathy, concluding that there is a comparable and even better effect than conventional treatment, with fewer undesirable reactions. 

 Considering these harmful effects arising from the treatment of anxiety and depression disorders through the use of conventional medications, Grimaldi-Bensouda et al.^27^ concluded, after comparing the use of conventional psychotropic drugs, the regular use of homeopathic medicines alone, and the mixed use of these two types of medicine among patients seeking care for anxiety and depression disorders, that in addition to homeopathy with the possibility of helping with the rebound effect of these drugs, patients treated with homeopathy, exclusively or in combination, were less likely to use psychotropic drugs over 12 months compared to those treated conventionally, and the rate of clinical improvement was higher for the group treated exclusively with homeopathy than for those treated with conventional treatment. 

 In this study, comparing the scores obtained by applying the questionnaires at the first appointment and 3 and 6 months after the start of homeopathic treatment with regard to the quality of sleep and the levels of stress, anxiety, and depression, a statistically significant improvement was observed in these four variables after 6 months of homeopathic treatment (Figure 2). 

 Data of the correlation of sleep quality with the level of stress, anxiety, and depression (Table 2) show that both stress and anxiety, to a greater extent, interfere with the quality of sleep, which, in a way, also corroborates epidemiological studies that show that sleep disorders, particularly insomnia, affect approximately 50% of individuals with anxiety.^28^

 It is important to highlight that the treatment was not only aimed at complaints regarding sleep and/or emotional state, as the choice of the most appropriate homeopathic medicine for each individual case followed the Law of Similars, which is one of the laws that rule homeopathy, considering the individual’s totality of presented symptoms, which is another principle of medical rationality. 

 The results described here could be even more significant if the study time is longer and the number of individuals evaluated is greater. The absence of the 74 individuals initially assessed after six months may have compromised the representativeness of the sample and the robustness of the conclusions. 

 The fact that questionnaires can be considered long and may have made the consultation convenient for the patient and for the doctor who administered the questionnaires, and recall bias can all be seen as potential limitations of this study. 

 The homeopathic care offered in just two locations of the city (difficult to access for many residents) may have contributed significantly to absenteeism as well as economic conditions, both in terms of the patients’ ability to travel to care locations and in the purchase of homeopathic medicines, representing a financial barrier that may have affected adherence to treatment and, consequently, the results of the study. 

 Due to these considerations, difficulties were observed in the municipality in relation to both geographic and economic accessibility. Furthermore, the centralization of homeopathic care in just two healthcare services in a large municipality (with an area of 319,19 km^2^) and the non-availability of homeopathic medicines free of charge are still negative aspects of this scenario, which compromises and hinders the effectiveness of homeopathic treatment.^29-30^

 Based on the acquired results, homeopathic treatment may help prevent risk factors for NCDs, as the improvement in sleep quality and levels of stress, anxiety, and depression was evident and significant after 6 months of individualized homeopathic medical follow-up in the studied population. 

## CONCLUSION

 Homeopathic treatment had a positive impact on the studied population, with improvements in sleep quality and levels of stress, anxiety, and depression in a short period (3–6 months), suggesting that this therapy could be used as a prevention strategy for NCDs. 
